# Impact of Microbiota Transplant on Resistome of Gut Microbiota in Gnotobiotic Piglets and Human Subjects

**DOI:** 10.3389/fmicb.2020.00932

**Published:** 2020-05-19

**Authors:** Hu Liu, Hua H. Wang

**Affiliations:** ^1^State Key Laboratory of Animal Nutrition, College of Animal Science and Technology, China Agricultural University, Beijing, China; ^2^Department of Food Science and Technology, The Ohio State University, Columbus, OH, United States; ^3^Department of Microbiology, The Ohio State University, Columbus, OH, United States

**Keywords:** gut resistome, microbiota transplant, vaginal seeding, gut microbiota dysbiosis, oral antibiotic administration, gut impacting drugs, antibiotic resistance genes

## Abstract

Microbiota transplant is becoming a popular process to restore or initiate “healthy” gut microbiota and immunity. But, the potential risks of the related practices need to be carefully evaluated. This study retrospectively examined the resistomes of donated fecal microbiota for treating intestinal disorders, vaginal microbiota of pregnant women, and infant fecal microbiota from rural and urban communities, as well as the impact of transplants on the fecal resistome of human and animal recipients. Antibiotic resistance (AR) genes were found to be abundant in all donor microbiota. An overall surge of resistomes with higher prevalence and abundance of AR genes was observed in the feces of all transplanted gnotobiotic pigs as well as in the feces of infant subjects, compared to those in donor fecal and maternal vaginal microbiota. Surprisingly, transplants using rural Amish microbiota led to more instead of less AR genes in the fecal microbiota of gnotobiotic pigs than did transplants using urban microbiota. New AR gene subtypes undetected originally also appeared in gnotobiotic pigs, in Crohn’s Disease (CD) patients after transplant, and in feces of infant subjects. The data illustrated the key role of the host gastrointestinal tract system in amplifying the ever-increasing AR gene pool, even without antibiotic exposure. The data further suggest that the current approaches of microbiota transplant can introduce significant health risk factor(s) to the recipients, and newborn human and animal hosts with naïve gut microbiota were especially susceptible. Given the illustrated public health risks of microbiota transplant, minimizing massive and unnecessary damages to gut microbiota by oral antibiotics and other gut impacting drugs becomes important. Since eliminating risk factors including AR bacteria and opportunistic pathogens directly from donor microbiota is still difficult to achieve, developing microbial cocktails with defined organisms and functions has further become an urgent need, should microbiota transplantation become necessary.

## Introduction

Human and animal gastrointestinal (GI) tracts are ecosystems colonized with trillions of diverse microbes and have a level of complexity comparable to that of an organ system (Kelly et al., 2015). The host gut and its microbiome have coevolved as a physiological community (Aagaard et al., 2014). Gut microbes have important roles in host health, from nutrient absorption to modulating the functions of the host immune system and the gut-brain interactions (Chung et al., 2012; Flint et al., 2012; Liu et al., 2018). While the exact mechanisms and pathways involved in the complex interplay between hosts and the gut microbiota still remain largely unknown, gut microbiota dysbiosis has been associated with various “modern” human health problems, such as inflammatory bowel diseases (IBDs), type 2 diabetes, colorectal cancer in adults (Ferretti et al., 2018), *Clostridium difficile* infection (CDI), necrotizing enterocolitis, asthma in children (Khanna et al., 2013; Moos et al., 2016), mental health disorders (Rieder et al., 2017), and even affecting the efficacy of drugs (Viswanathan, 2013; Gopalakrishnan et al., 2018; Pinato et al., 2019). In the past few years, microbiota transplant (MT) has emerged as a popular strategy to replenish healthy gut microbiota and treat diseases (Khanna, 2018). For example, fecal microbiota transplant (FMT) is found effective in treating CDI and eliminating *C. difficile* from the intestinal tract of infected patients (Leung et al., 2018). Seeding maternal vaginal microbiota to infant mouth and skin of Cesarean section (C-section) delivered babies has also been practiced with the intention to help these babies develop normal gut microbiota (Dominguez-Bello et al., 2016). However, emerging evidences also illustrated that public health risk factors, such as viruses or bacterial pathogens, were introduced to patients’ microbiota through MT (Huynh et al., 2018; Tan and Johnson, 2019). Despite FMT being presented as a potential mechanism to reduce gut resistome, the data from Leung et al. (2018) in fact clearly demonstrated that FMT introduced new antibiotic resistant (AR) genes to the patients.

The rapid surge of AR pathogens has become a top global public health threat (Zhang et al., 2013; Laffin et al., 2017; World Health Organization, 2019). However, effective mitigation only becomes possible when our scope and strategies are no longer restricted to limiting the applications of antibiotics (Doyle et al., 2006; Wang, 2010; Wang and Schaffner, 2011; Millan et al., 2016). For instance, once AR commensal bacteria and even probiotics and fermentation starter cultures associated with ready-to-consume foods were identified as the key avenue impacting host gut resistome through food intake, successful mitigation of the largest foodborne AR gene pool was achieved in just 4 years (2006–2010), owing to the quick removal of the problematic starter cultures and probiotics from the product lines (Li et al., 2011). Thus, redefining probiotics and mandatory safety screening of microbes intended for beneficial applications across human health as well as food animal and agricultural productions have also become stressing needs in order to mitigate the spread AR in the ecosystem.

The host gastrointestinal tract system has pivotal roles in AR ecology independent from antibiotic exposure. Without conventional food exposure, antibiotic resistance genes (ARGs), and resistant bacteria were still found quickly surged in infant fecal microbiota within days after birth without antibiotic treatment, most likely due to the oral/nasal exposure to mother’s microbiota during natural birth and breastfeeding (Zhang et al., 2011). ARGs and resistant bacteria are also found abundant in feces of animals from antibiotic-free production system (Cottell et al., 2012; Zhou, 2016). Zhang et al. (2013) further illustrated that 5-day antibiotic treatment only led to the rapid surge of the targeted ARGs in fecal microbiota of mice previously seeded with the corresponding ARG-containing bacteria, but not in placebo mice without seeding, regardless of the 5-day antibiotic treatment. These data clearly illustrated that, besides the application of antibiotic itself, introducing exogenous AR bacteria to the host GI tract system is an overlooked, but key risk factor leading to the rapid surge of AR bacteria in the gut and fecal microbiota. Unfortunately, the public health risk has already turned into a reality. According to the safety alert by the U.S. Food and Drug Administration (FDA) issued on June 13, 2019, two immunocompromised adults contracted invasive infections from FMT that contained extended-spectrum β-lactamase (ESBL)-producing *Escherichia coli* (The Food and Drug Administration (FDA), 2019; DeFilipp et al., 2019). One of these patients died. Therefore, improving knowledge as well as communicating and further limiting the public health consequences of MT have become critical.

In recent years, high-throughput sequencing (HTS)-based metagenomics have revolutionized our understanding of host microbiome, especially the complexity and diversity of the gut microbiota (Speth and Orphan, 2018). The availabilities of the metagenomics analysis tools (Chen et al., 2019) as well as the abundance of gut microbiome sequence data from various studies have made investigations with new scope possible. By retrospectively reassessing cohorts of metagenomics datasets, this study revealed the dynamics of resistomes in recipients associated with MT, and the potential public health risks associated with related practices.

## Materials and Methods

### Sample Information

Recently published shotgun metagenomes from stool samples of infants, patients with Crohn’s Disease (CD), vaginal samples from mothers before delivery, as well as transplanted piglets were targeted to assess the impact of MT on AR dissemination and colonization in the gut of recipients. Raw metagenomics data sets were filtered according to the following criteria for further analyses: (1) the samples should be related to MT; (2) the shotgun metagenomics data have been previously published and are available; and (3) the samples provided sufficient background information (such as donors and recipients).

Metagenomics data of a total of 44 samples resulting from 3 independently published studies were used in this study. Samples included 6 maternal vaginal samples, and stools of 16 infants, 10 CD patients, 4 donors, and 8 humanized germ-free piglets (Ferretti et al., 2018; Vaughn et al., 2016; Dhakal et al., 2019).

### Metagenomics Data Retrieval and Preprocessing

The Sequence Read Archive database (SRA) was retrieved from the repository NCBI_SRA (BioProject Accession: PRJNA484151, Table 1; PRJNA321058, Table 2; and PRJNA352475, Table 3) in.sra format. The retrieved files were converted to.fastq using SRA Toolkit 2.9.6 for Ubuntu Linux^1^. Tables 1–3 summarized the sequence information. A series of independent data-“dump” utilities were included in the SRA Toolkit, which were used to convert SRA data to different file formats. For instance, Fastq-dump allowed for conversion of SRA data into fastq or fasta format in the current study. The FASTP tool (v0.19.5) was used for quality control (QC) and removal of low quality sequences with default parameters before annotation and analysis of the generated raw reads in each metagenomics dataset (Chen et al., 2018). The parameters of filtering data by FASTP tool including: phred quality of reads ≤ Q15 were unqualified and removed; 40% of bases in reads are allowed to be unqualified; sequences with five or more non-ATCG characters were also removed; reads shorter than required length of 80 were discarded.

**TABLE 1:** The sequence accession numbers in the list of Bioproject PRJNA484151.

Run	BioSample	AvgSpotLen	Collection_date	Experiment	Isolation_source	Library name	MBases	MBytes	Organism	Sample name
SRR7642163	SAMN09762371	474	1-June-15	SRX4505614	Homo sapiens-Amish infant2	HR6	554	319	Human metagenome	HR6
SRR7642164	SAMN09762379	466	1-Jun-15	SRX4505613	Homo sapiens-Non-Amish infant5	HU18	997	568	Human metagenome	HU18
SRR7642165	SAMN09762378	466	1-Jun-15	SRX4505612	Homo sapiens-Non-Amish infant4	HU17	1019	599	Human metagenome	HU17
SRR7642166	SAMN09762370	468	1-Jun-15	SRX4505611	Homo sapiens-Amish infant1	HR5	904	544	Human metagenome	HR5
SRR7642167	SAMN09762381	467	1-Aug-16	SRX4505610	Sus scrofa domesticus2	PUC2	759	439	Pig metagenome	PUC2
SRR7642168	SAMN09762380	467	1-Aug-16	SRX4505609	Sus scrofa domesticus1	PUC1	915	574	Pig metagenome	PUC1
SRR7642169	SAMN09762383	474	1-Aug-16	SRX4505608	Sus scrofa domesticus4	PUC4	599	357	Pig metagenome	PUC4
SRR7642170	SAMN09762382	463	1-Aug-16	SRX4505607	Sus scrofa domesticus3	PUC3	808	461	Pig metagenome	PUC3
SRR7642171	SAMN09762385	465	1-Dec-15	SRX4505606	Sus scrofa domesticus6	PRC2	775	449	Pig metagenome	PRC2
SRR7642172	SAMN09762384	467	1-Dec-15	SRX4505605	Sus scrofa domesticus5	PRC1	740	441	Pig metagenome	PRC1
SRR7642173	SAMN09762387	468	1-Dec-15	SRX4505604	Sus scrofa domesticus8	PRC4	798	490	Pig metagenome	PRC4
SRR7642174	SAMN09762386	464	1-Dec-15	SRX4505603	Sus scrofa domesticus7	PRC3	843	503	Pig metagenome	PRC3
SRR7642175	SAMN09762376	463	1-Jun-15	SRX4505602	Homo sapiens-Non-Amish infant2	HU12	901	514	Human metagenome	HU12
SRR7642176	SAMN09762373	451	1-Jun-15	SRX4505601	Homo sapiens-Amish infant5	HR8	761	442	Human metagenome	HR8
SRR7642177	SAMN09762377	467	1-Jun-15	SRX4505600	Homo sapiens-Non-Amish infant3	HU16	812	484	Human metagenome	HU16
SRR7642178	SAMN09762375	470	1-Jun-15	SRX4505599	Homo sapiens-Non-Amish infant1	HU11	690	404	Human metagenome	HU11
SRR7642179	SAMN09762372	472	1-Jun-15	SRX4505598	Homo sapiens-Amish infant3	HR7	712	399	Human metagenome	HR7
SRR7642180	SAMN09762374	474	1-Jun-15	SRX4505597	Homo sapiens-Amish infant4	HR10	675	385	Human metagenome	HR10

**TABLE 2:** The sequence accession numbers in the list of Bioproject PRJNA321058.

Run	BioSample	AvgSpotLen	Experiment	Gastroinstest_disord	Host_subject_ID	Library name	MBases	MBytes	Sample name	Time_point
SRR3582147	SAMN05065750	197	SRX1797372	Crohn’s disease	R1009	G85458	2073	1394	G85458	pre-FMT
SRR3582154	SAMN05065730	198	SRX1797379	Crohn’s disease	R1007	G85435	3298	2224	G85435	pre-FMT
SRR3582173	SAMN05065774	197	SRX1797398	Crohn’s disease	R1022	G85485	3183	2287	G85485	pre-FMT
SRR3582175	SAMN05065776	198	SRX1797400	Crohn’s disease	R1021	G85487	2925	1989	G85487	pre-FMT
SRR3582180	SAMN05065733	198	SRX1797405	Crohn’s disease	R1005	G85438	3398	2294	G85438	pre-FMT
SRR3582138	SAMN05065742	198	SRX1797363	Crohn’s disease	R1005	G85450	2170	1463	G85450	4 weeks after FMT
SRR3582139	SAMN05065743	198	SRX1797364	Crohn’s disease	R1007	G85451	3172	2129	G85451	4 weeks after FMT
SRR3582148	SAMN05065751	199	SRX1797373	Crohn’s disease	R1009	G85459	5387	3677	G85459	4 weeks after FMT
SRR3582170	SAMN05065771	199	SRX1797395	Crohn’s disease	R1022	G85482	2948	2010	G85482	4 weeks after FMT
SRR3582172	SAMN05065773	198	SRX1797397	Crohn’s disease	R1021	G85484	2149	1437	G85484	4 weeks after FMT
SRR3582142	SAMN05065746	198	SRX1797367	NA	D9004	G85454	4279	2888	G85454	NA
SRR3582134	SAMN05065738	199	SRX1797359	NA	D9001	G85444	4639	3132	G85444	NA
SRR3582179	SAMN05065779	199	SRX1797404	NA	D9002	G90607	9475	3633	G90607	NA
SRR3582158	SAMN05065760	199	SRX1797383	NA	D9005	G85470	5901	3980	G85470	NA

**TABLE 3:** The sequence accession numbers in the list of Bioproject PRJNA352475.

Run	BioSample	AvgSpotLen	Couple_Id	Donor	Experiment	Infant_age	Isolation_source	Library name	MBases	MBytes	Sample name
SRR5273969	SAMN06350145	181	10031	Mother	SRX2577952	Not applicable	Vaginal introitus	2527_t0	2359	1085	CA_C10031MS2527VA_t0M15
SRR5273966	SAMN06350148	197	10031	Infant	SRX2577949	3 days	Stool	2537_t3	2243	1054	CA_C10031IS2537FE_t3M15
SRR5274002	SAMN06350112	192	10023	Mother	SRX2577985	Not applicable	Vaginal introitus	2387_t0	1251	568	CA_C10023MS2387VA_t0M15
SRR5274004	SAMN06350110	183	10023	Infant	SRX2577987	3 days	Stool	2393_t2	4984	2246	CA_C10023IS2393FE_t2M15
SRR5273911	SAMN06350203	195	10043	Mother	SRX2577894	Not applicable	Vaginal introitus	2743_t0	786	352	CA_C10043MS2743VA_t0M15
SRR5273915	SAMN06350199	195	10043	Infant	SRX2577898	3 days	Stool	2749_t2	5267	2373	CA_C10043IS2749FE_t2M16
SRR5273991	SAMN06350123	174	10024	Mother	SRX2577974	Not applicable	Vaginal introitus	2405_t0	198	87	CA_C10024MS2405VA_t0M15
SRR5273995	SAMN06350119	181	10024	Infant	SRX2577978	3 days	Stool	2411_t2	5175	2339	CA_C10024IS2411FE_t2M15
SRR5273932	SAMN06350182	188	10039	Mother	SRX2577915	Not applicable	Vaginal introitus	2671_t0	134	60	CA_C10039MS2671VA_t0M15
SRR5273929	SAMN06350185	194	10039	Infant	SRX2577912	3 days	Stool	2677_t2	3873	1791	CA_C10039IS2677FE_t2M16
SRR5274056	SAMN06350058	182	10007	Mother	SRX2578039	Not applicable	Vaginal introitus	2099_t0	167	73	CA_C10007MS2099VA_t0M15
SRR5274053	SAMN06350061	179	10007	Infant	SRX2578036	3 days	Stool	2105_t2	2885	1310	CA_C10007IS2105FE_t2M15

### Relative Abundance of ARGs

To characterize the profiles of ARGs, a direct annotation service was employed by uploading the post-QC processing paired-end metagenome reads to the DeepARG web service^2^. The DeepARG web service contains a fully automated raw metagenomic analysis pipeline for ARG annotation with a high degree of confidence (Arango-Argoty et al., 2018). This tool is based on the deepARG algorithm and the deepARG-DB, which currently comprises 30 antibiotic categories, 2149 groups, and 14,933 non-redundant reference sequences (2203 from CARD, 10,602 from UNIPROT, and 2128 from ARDB), and has been under continuous inspection (Arango-Argoty et al., 2018). The results of ARG profiles are normalized to the 16S rRNA gene abundance in the samples automatically by the platform, based on the deepARG algorithm and the deepARG-DB, as described at the DeepARG website^3^.

### Statistical Analysis

The datasets of the three studies were analyzed independently. The relative abundance of ARGs were processed in R designed by R Core Team (version 3.5.2) using the package ggplot2 (version 3.1.0). To distinguish the differences in diversity and abundance of the types of ARGs, boxplots of each detected ARG class with significance levels were visualized using packages ggpubr (version 0.2.4) in R software. The ARG subtypes of the pairing samples to compare with Venny 2.1^4^ to generate the Venn diagram.

## Results and Discussion

### Rural and Urban Infant Fecal Microbiota Transplant

**FIGURE 1:**
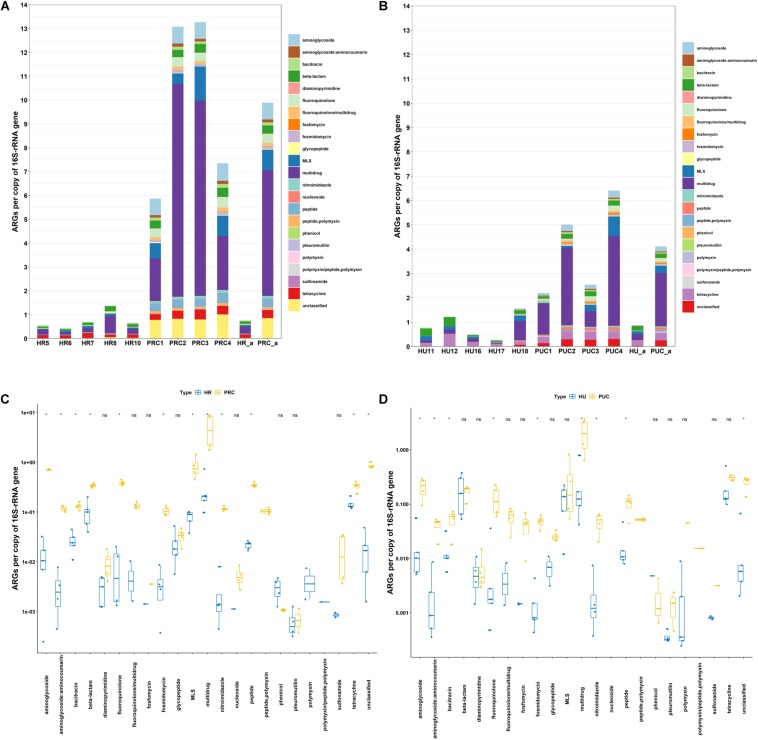
Abundance of ARGs in host gut microbiota. **(A)** Fecal microbiota of rural infants and transplanted piglets. **(B)** Fecal microbiota of urban infants and transplanted piglets. **(C)** Comparison of fecal microbiota of rural infants and transplanted piglets. **(D)** Comparison of fecal microbiota of urban infants and transplanted piglets. HR, rural infant; HU, urban infant; PRC, transplanted piglets from rural infant; PUC, transplanted piglets from urban infant; a, average value.

The relative abundance of ARGs varied from 0.43 to 1.37 in the rural infant stools, and 0.26 to 1.56 in urban infant stools (Figures 1A,B). These results illustrated that the abundances of ARGs in the rural and urban infant stools were quite similar. In total, 22 classes of ARGs were found in all samples. Multidrug resistance genes were the most abundant ARGs in the rural and urban infant stools, followed by those encoding resistance to tetracycline, β-lactam, and macrolide-lincosamide-streptogramin (MLS). The high abundance of these four most dominant AR types is consistent with previous reports of fecal samples from different countries (Yang et al., 2016; Feng et al., 2018). The differences between the abundance of each ARG type in the two types of samples were also analyzed. Among the 22 ARG types, the abundance of each ARG type exhibited no significant difference between the two types of samples.

Clearly, the profiles of human gut microbiome are influenced by many factors associated with rural or urban lifestyle (Leszczyszyn et al., 2016). Previously, several studies have compared rural and urban gut microbiota, and proposed that rural communities can serve as a source of preferred healthy microbiota. For instance, Tyakht et al. (2014) suggested that transplantation using fecal microbiota from rural communities may be an effective way to restore healthy gut microbiota in the human population of industrialized countries. However, data from this study revealed similar abundance of ARGs in fecal microbiota of rural Amish and urban infants. Although the initial intention of Dhakal et al. (2019) who generated the metagenomics data used in this study was to illustrate the potential of FMT in enhancing host immune function, our fecal resistome results clearly demonstrated that fecal microbiota of rural (Amish) community had comparable ARGs to those of urban subjects, and therefore no less health risk.

Figures 1A,B also illustrated that the relative abundance of ARGs varied from 5.86 to 13.27 in the rural fecal transplanted piglets, and 2.19 to 6.41 in piglets transplanted with urban fecal microbiota. In total, 23 classes of ARGs were found in all samples. The genes resistant to multidrug, unclassified, MLS, aminoglycoside, and fluoroquinolone were the five most dominant types in piglets transplanted with rural fecal microbiota. However, the most abundant ARGs in piglets transplanted with urban fecal microbiota were those encoding resistance to multidrug, tetracycline, MLS, unclassified, and aminoglycoside. The data suggested that even when the two groups of donors had comparable AR gene prevalence and abundance, the fecal microbiota profiles and resistomes of the transplanted pigs corresponding to rural, and urban donors varied significantly. Instead of being safer and healthier source of microbiota, as shown in Figure 1, the relative abundance of aminoglycoside, β-lactam, fluoroquinolone, nitroimidazole, and peptide resistance genes in piglets transplanted with rural fecal microbiota were actually considerably higher than those in piglets transplanted with urban fecal microbiota.

The impact of the resistomes of donors on that of the recipients was further illustrated by the relative abundance of ARGs in samples of rural infants and the transplanted piglets (Figure 1A). The abundance of ARGs in piglets transplanted with rural fecal microbiota was on average 13.1 times higher than that in the rural infant donor stools. Similarly, the abundance of ARGs in piglets transplanted with urban fecal microbiota was on average 4.7 times greater than those in the corresponding urban infant stool (Figure 1B). Among the 23 ARG types, the abundance of aminoglycoside, β-lactam, fluoroquinolone, MLS, and multidrug resistance genes in gut microbiota of the transplanted piglets were significantly higher than those in the infant donor stools (Figures 1C,D). The significant amplification of the ARGs in germ-free recipient pigs suggested that the impact of the microbial inoculation is especially prominent in recipients with naïve gut microbiota. These results in germ-free piglet models were consistent with previous studies concluding that the gut microbiota of infants had higher abundance of ARGs compared to those of their mothers, even in the absence of antibiotic exposure (Parnanen et al., 2018).

In total, 141 and 173 ARG subtypes were detected in 5 rural infants and 4 piglets transplanted with rural fecal microbiota, respectively (Figure 4A). Out of the 193 subtypes of ARGs detected in all samples, a total of 52 subtypes were only detected in piglets transplanted with rural fecal microbiota, accounting for 26.9% of total number of ARG subtypes detected. Similarly, the ARG subtypes detected in 5 urban infants and 4 transplanted piglets were 141 and 170, respectively. The unique ARG subtypes in 4 transplanted piglets were 42, contributing to 23.0% of the total ARG subtype numbers detected.

Previous studies have shown that the pre-existing gut microbiota significantly affected the outcomes of MT (Ross et al., 2019). But the rapid surge of ARGs in the gut microbiota of naïve pig recipients after MT, especially the difference between the recipients and the original donors, were still astonishing and need special attention. This finding suggests that even without antibiotic exposure, seeding of AR bacteria to naïve GI tract systems may have been a critical contributor to the rapid enrichment of certain AR bacteria in human and animal feces, including the emergence of ARGs previously below detection limit in the donor microbiome, further impacting the environmental AR gene pool. Moreover, AR bacteria from rural infant donors led to higher proliferation of ARGs in fecal microbiota of the recipients than those of urban infants in this case. Whether this impact is due to the long-term fitness of such bacteria in hosts, despite rural populations rarely exposed to modern drugs, is yet to be elucidated.

### Resistome of Maternal Vaginal Microbiota and the Potential Impact on Infants

**FIGURE 2:**
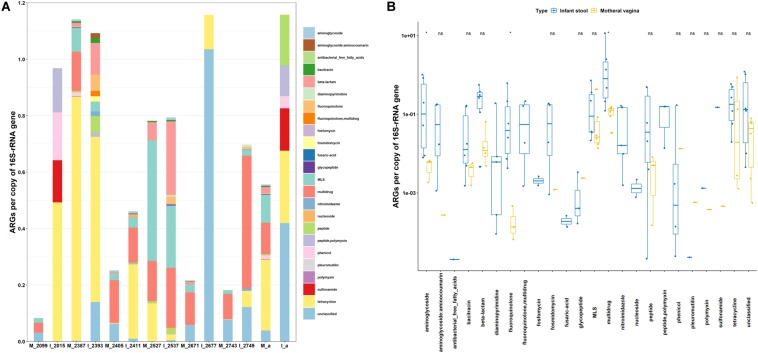
Abundance of ARGs in host gut microbiota. **(A)** Vaginal microbiota of expectant mother and fecal microbiota of her infant. **(B)** Comparison of vaginal microbiota of expectant mother and fecal microbiota of her infant. M, mother; I, infant; a, average value. The number is Library Name in the list of Bioproject PRJNA352475.

To access the potential health risks of vaginal seeding beyond the possible transmission of pathogens and viruses (Huynh et al., 2018; Simpson, 2018), the resistomes of six maternal vaginas were examined in this study and illustrated in Figure 2. The relative abundance of ARGs varied from 0.08 to 1.14 in vaginal microbiota. In total, 16 classes of ARGs were found in all samples (Figure 2A). The resistance genes to tetracycline, multidrug, MLS, unclassified, and β-lactam were the five most dominant types in maternal vaginas. The comparisons of ARG abundance between maternal vagina and the corresponding infant stool samples were shown in Figures 2A,B. The abundance of aminoglycoside, fluoroquinolone, and multidrug resistance genes in infant stools were significantly higher than those in the corresponding maternal vaginas. Moreover, out of the 24 classes of ARGs, the abundance of fosfomycin, nucleoside, and diaminopyrimidine could only be found in infant stool samples, which were below the detection limit in maternal vaginal microbiota. The data are consistent with the findings from the transplanted pigs.

The significant enrichment and expanding of the ARG profiles in infants, in agreement with the results from germ-free recipient pigs, also suggested that the impact of the initial microbial inoculation is especially prominent in recipients with naïve gut microbiota. The data indicated the potential risk of transmitting ARGs through exposure to vaginal or fecal microbiota, which is further in agreement with previous conclusions that bacterial communities acquired by vaginally delivered infants resembled their own mother’s vaginal microbiota (Dominguez-Bello et al., 2010), and the early development of ARGs in the infant gut microbiota is likely impacted by exposure to maternal and environmental microbes (Zhang et al., 2011). It is worth noting that while the germ-free piglets shared the same delivery approach, sanitized living environment, and heat-treated dairy feed, human infants were exposed to more environmental and feeding variables, through environmental contact, breastfeeding, etc.

### Shifts in ARG Abundance in Crohn’s Disease Patients Following Fecal Microbiota Transplant

**FIGURE 3:**
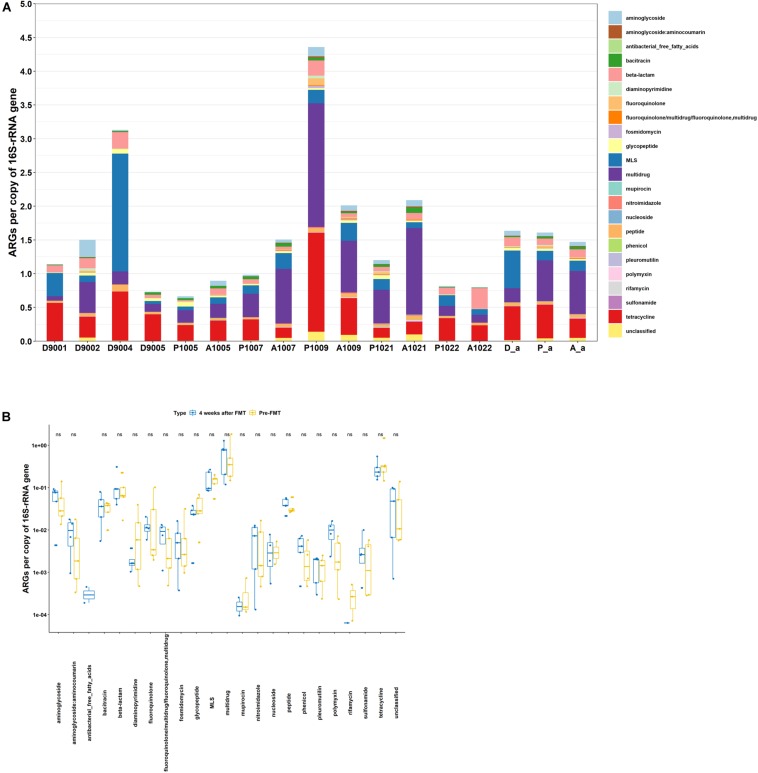
Abundance of ARGs in host gut microbiota. **(A)** Fecal microbiota of donors, Crohn’ Disease (CD) patients pre-and post-FMT. **(B)** Comparison of fecal microbiota of CD patients pre-and post-FMT. D, donor; P, pre-FMT; A, post-FMT; a, average value. The number is host_subject_ID in the list of Bioproject PRJNA321058.

As shown in Figure 3A, the abundance of ARGs varied from 0.66 to 4.36 and 0.80 to 2.09 in fecal microbiota of CD patients pre- FMT and 4 weeks post- FMT, respectively. The average abundance of ARGs in patients pre-FMT and 4 weeks post-FMT were quite similar. However, the total ARG abundance after FMT increased in 3 out of 5 patients, remained about the same in one patient, and decreased in another patient (Figure 3A). Meanwhile, the top five most abundant ARGs in patients of both pre-FMT as well as 4 weeks post-FMT were multidrug, tetracycline, MLS, β-lactam, and aminoglycoside (Figure 3B). Among the 22 ARG types, the abundance of each ARG type exhibited no significant difference between the two types of samples (Figure 3B).

**FIGURE 4:**
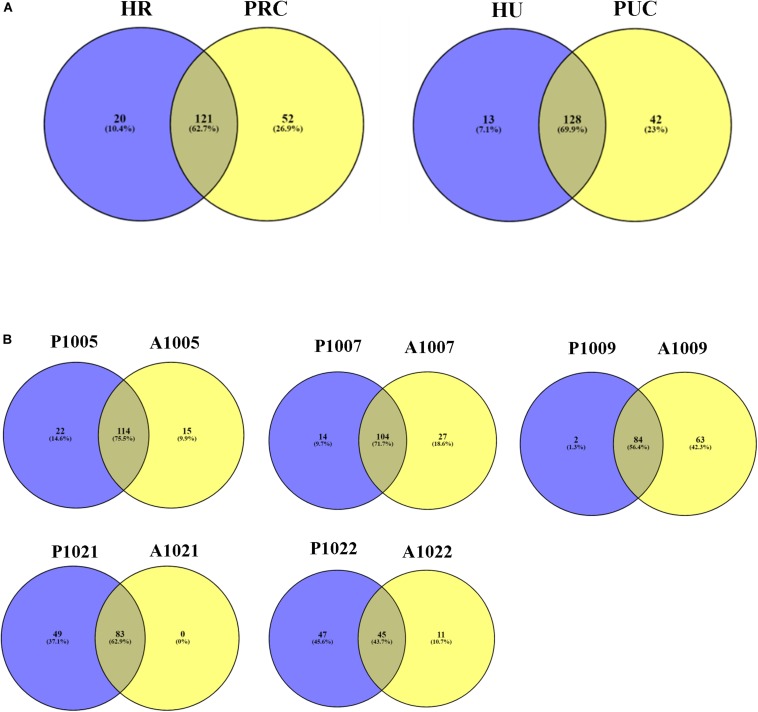
Venn diagram showing the number of shared and unique ARGs. **(A)** Fecal microbiota of infants and transplanted piglets. **(B)** Fecal microbiota of Crohn’s Disease (CD) patients pre-and post-FMT. Resistome of fecal microbiota of Crohn’s Disease patients pre- and post-FMT. HR, rural infant; HU, urban infant; PRC, transplanted piglets from rural infant; PUC, transplanted piglets from urban infant; P, pre-FMT; A, post-FMT. The number is host_subject_ID in the list of Bioproject PRJNA321058.

The number of detected ARG subtypes in stool samples of patient recipients varied from 103 to 151 in pre-FMT and 4 weeks post-FMT (Figure 4B). Out of the total ARG subtypes detected in all samples, the unique ARG subtypes varied from 11 to 63 in stool samples of recipients 4 weeks post-FMT, accounting for 9.9–42.3% of the total ARG subtypes detected. The stool sample of recipient A1021 (Accession number: SRR3582172) was an exception, without unique ARG subtypes being detected.

Despite several reports proposed that FMT can be effective in eradicating pathogenic AR bacteria and ARGs, data also showed that several clinically significant ARGs emerged in recipient gut microbiota immediately after FMT (Kao et al., 2016; Millan et al., 2016; Leung et al., 2018). Our findings are in agreement with previous reports showing that the pre-existing gut microbiota significantly affected the outcomes of MT (Ross et al., 2019) and even *C. difficile* infection could still reoccur after FMT (Millan et al., 2016). In this study, the increase, retention, and decrease of abundance of ARGs were 60, 20, and 20%, respectively, in patients 4 weeks after FMT. It is expected that the responses to FMT were related to the existing gut microbiota of the recipients, which might vary among subjects.

## Conclusion and Perspective

The retro-assessments of the metagenomics data from 3 different studies clearly demonstrated that ARGs are prevalent across multiple body habitats, i.e., guts, vagina, etc., as well as in rural and urban populations, and MTs can spread risk factors including AR bacteria to the recipients. The rapid surge of ARGs in transplanted piglets and infants that far exceeded the abundances found in the donor microbiota revealed the potential public health risks associated with MTs, especially to subjects with naïve gut microbiota. This finding also confirmed another overlooked key cause of the escalated swelling of ARGs in the next generation of hosts—with history of no exposure to antibiotic but surely to AR bacteria via natural birth, breastfeeding, conventional eating, and waste contamination, etc.

While replenishing the gut with healthy microbiota is a good intention, the term “healthy microbiota” is far more complicated. The FDA clearly discouraged MT but supported the uses of defined strains in 2016. Expanding cocktails of defined strains likely can be a practical approach leading to more productive outcomes.

Fecal microbiota transplant had varied impact on the gut microbiota of adult CD patients, reflecting the impact of recipients’ existing microbiota on the newcomers. Oral drugs, drugs excreted through the liver/gut, and especially oral administration of antibiotics massively destroy existing gut microbiota (Shen et al., 2011; Zhang et al., 2013; Liang et al., 2015; Isaac et al., 2017; Maier et al., 2018; Maseda et al., 2019; Morjaria et al., 2019), not only selecting resistant bacteria and leading to gut microbiota dysbiosis, but also creating a new naïve gut environment that potentially facilitates the surge of pathogens and ARGs in patients due to the lack of effective defense from the existing gut microbiota.

In conclusion, MT is still a practice with significant public health risk. It may potentially be used as the last option to treat severe diseases instead of a common practice intended to improve host health. Proper strategies to retain and improve gut health should rely on minimizing unnecessary damages to gut microbiota, such as massive AR and gut microbiota dysbiosis due to oral administration of antibiotics and taking gut impacting drugs. While eliminating risk factors including AR bacteria and opportunistic pathogens directly from donor fecal microbiota is still hard to achieve, developing microbial cocktails with defined organisms and functions has become a stressing need should microbiota transplantation become necessary.

## Data Availability Statement

All datasets generated for this study are included in the article/supplementary material.

## Ethics Statement

The studies involving animal and human participants were reviewed and approved by the appropriate committees of the corresponding institutes. Written informed consent for participation was not required for this study in accordance with the National Legislation and the Institutional requirements.

## Author Contributions

HL and HW designed the research. HL executed the experiments, analyzed the data, and wrote the draft of the manuscript. HW supervised the study, provided guidance on the purpose of the project, published studies used to build the conclusion, reviewed the results, and revised the manuscript. Both authors approved the final manuscript.

## Conflict of Interest

The authors declare that the research was conducted in the absence of any commercial or financial relationships that could be construed as a potential conflict of interest.

## Footnotes

1 https://www.ncbi.nlm.nih.gov/sra/docs/toolkitsoft/

2 https://bench.cs.vt.edu/deeparg_analyze/

3 https://bench.cs.vt.edu/deeparg

4 https://bioinfogp.cnb.csic.es/tools/venny/index.html
